# Deciphering Epitranscriptome: Modification of mRNA Bases Provides a New Perspective for Post-transcriptional Regulation of Gene Expression

**DOI:** 10.3389/fcell.2021.628415

**Published:** 2021-03-16

**Authors:** Suresh Kumar, Trilochan Mohapatra

**Affiliations:** ^1^Division of Biochemistry, ICAR-Indian Agricultural Research Institute, New Delhi, India; ^2^Indian Council of Agricultural Research, New Delhi, India

**Keywords:** epitranscriptomics, RNA modification, post-transcriptional regulation, 5-methylcytidine, *N*^6^-methyladenosine, RNA metabolism, mRNA methylation, central dogma

## Abstract

Gene regulation depends on dynamic and reversibly modifiable biological and chemical information in the epigenome/epitranscriptome. Accumulating evidence suggests that messenger RNAs (mRNAs) are generated in flashing bursts in the cells in a precisely regulated manner. However, the different aspects of the underlying mechanisms are not fully understood. Cellular RNAs are post-transcriptionally modified at the base level, which alters the metabolism of mRNA. The current understanding of epitranscriptome in the animal system is far ahead of that in plants. The accumulating evidence indicates that the epitranscriptomic changes play vital roles in developmental processes and stress responses. Besides being non-genetically encoded, they can be of reversible nature and involved in fine-tuning the expression of gene. However, different aspects of base modifications in mRNAs are far from adequate to assign the molecular basis/functions to the epitranscriptomic changes. Advances in the chemogenetic RNA-labeling and high-throughput next-generation sequencing techniques are enabling functional analysis of the epitranscriptomic modifications to reveal their roles in mRNA biology. Mapping of the common mRNA modifications, including *N*^6^-methyladenosine (m^6^A), and 5-methylcytidine (m^5^C), have enabled the identification of other types of modifications, such as *N*^1^-methyladenosine. Methylation of bases in a transcript dynamically regulates the processing, cellular export, translation, and stability of the mRNA; thereby influence the important biological and physiological processes. Here, we summarize the findings in the field of mRNA base modifications with special emphasis on m^6^A, m^5^C, and their roles in growth, development, and stress tolerance, which provide a new perspective for the regulation of gene expression through post-transcriptional modification. This review also addresses some of the scientific and technical issues in epitranscriptomic study, put forward the viewpoints to resolve the issues, and discusses the future perspectives of the research in this area.

## Introduction

**FIGURE 1:**
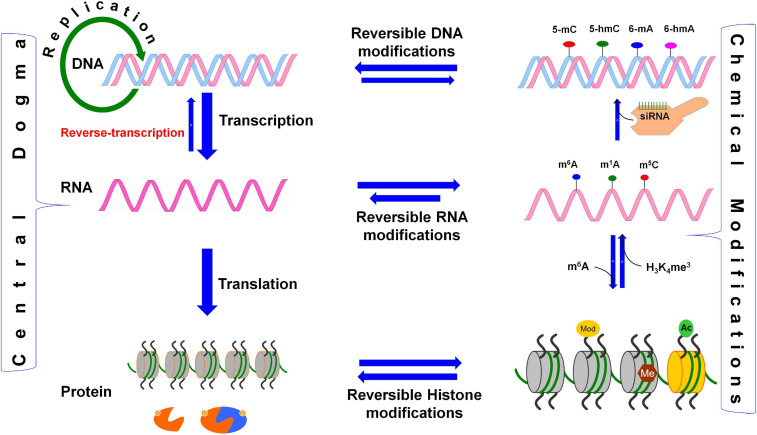
Reversible biochemical modifications affect the transfer of genetic information (the Central Dogma). As per the central dogma, the genetic information passes from DNA, through RNA, to protein. However, epigenetic DNA base modifications [e.g., 5-methylcytosine (5-mC), 5-hydroxymethylcytosine (5-hmC), *N*^6^-methyladenine (6-mA), and *N*^6^-hydroxymethyladenine (6-hmA)] and histone protein modifications [e.g., methylation (me) and acetylation (ac) of amino acids] affects RNA metabolism (including splicing, export, stability, and translation efficiency) and play crucial roles in the regulation of cellular growth, development, and protection from environmental stress. Similarly, the dynamic RNA modifications [e.g., *N*^6^-methyladenosine (m^6^A), *N*^1^-methyladenosine (m^1^A), and *N*^6^-hydroxymethyladenosine (hm^6^A)] encrypt an additional layer of information and dynamically regulate the biological processes. Small-interfering RNA (siRNA) plays important role in recruitment of DNA methyltranferase for DNA base modification, methylated mRNA bases (e.g., m^6^A) play role in protein synthesis, the histone 3 (H_3_) protein trimethylated (me^3^) at 4th lysine of (H_3_K_4_me^3^) affects the transcription process.

From the genome to proteome, several proficient biological processes regulate cellular growth and functions. Transcription of a gene is a truthful process, as the timing and rate of transcription are subjected to strict regulation, and its accuracy is vital for the vigor and development of the cell (Wang et al., 2018). Because translation of mRNA is a vital process in all living organisms, and assembly of the translational machinery followed by movement along the mRNA consumes ∼40% of cellular energy, the process needs to be precisely regulated to conserve energy. The ‘Central Dogma of life’ describes that genetic information is transformed from DNA to protein through RNA. Both DNA and histone proteins are reversibly modified (epigenetic modifications) to fine-tune the expression of genes/phenotypes (Fu Y. et al., 2014). An analogous process for RNA (epitranscriptomic modification) has been a missing component of the central dogma (Figure 1). Reversible biochemical modifications are known now to occur in most of the constituent processes of the central dogma, which dynamically control gene expression. The spectrum of epigenetic base modifications detected so far in DNA is relatively limited (six), about 170 distinct modifications have been identified in RNAs (Boccaletto et al., 2018; Kadumuri and Janga, 2018; Shen et al., 2019; Boo and Kim, 2020; Selmi et al., 2021). RNAs play vital roles in biological systems, not only as structural components [i.e., ribosomal RNAs (rRNAs)], translators [i.e., transfer RNAs (tRNAs)], and messengers (i.e., mRNAs, conveying genetic information to the protein) but also as regulators [i.e., small interfering RNAs (siRNAs), enhancer RNAs (eRNAs)] of several biological processes. The functions of rRNAs, tRNAs, and mRNAs are regulated through co- or post-transcriptional chemical modifications (Boccaletto et al., 2018; Boo and Kim, 2020), the exact role of many of these base modifications remain enigmatic. Although extensive base modifications in rRNAs and tRNAs in terms of the variety/abundance of modifications are well known and have remained undisputed for many decades (Jackman and Alfonzo, 2013), all other classes of RNA are subjected to enzymatic modifications (Xu L. et al., 2017). Several post-transcriptional base modifications in messenger RNA (mRNA) have only recently been identified. Such mRNA base modifications affect different cellular processes like pre-mRNA splicing, mRNA export, translation, and degradation, which shape the cellular transcriptome and proteome. Recent findings indicate that the level of proteins in a cell does not necessarily correspond with the mRNA level (Khan et al., 2013; Wu et al., 2013), which might vary because of various post-transcriptional regulation, including epitranscriptomic modifications affecting mRNA biology. The recent advances in experimental techniques have facilitated the identification of different epitranscriptomic modifications in the coding and untranslated regions (UTRs) of mRNAs (Zhao et al., 2020). While the functions of some of the epitranscriptomic modifications are known, occurrence and function of many other diverse epitranscriptomic modifications are still to be established.

**FIGURE 2:**
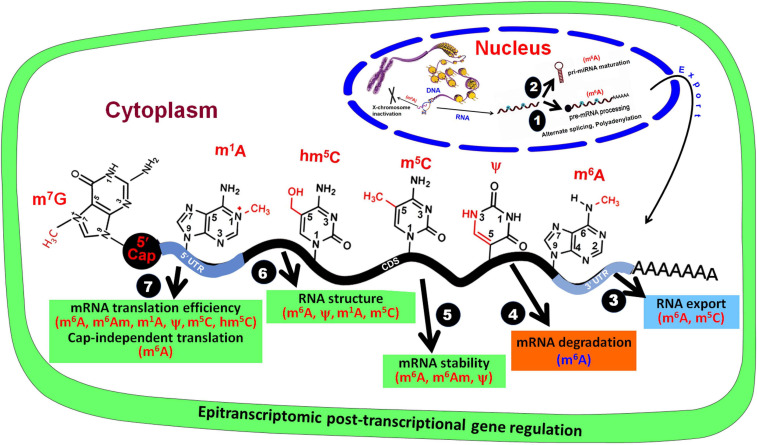
Base modifications in mRNA affect post-transcriptional gene regulation. In the nucleus, RNA base modifications affect (1) pre-mRNA processing and (2) pri-miRNA maturation, and (3) their export from the nucleus. In the cytoplasm, RNA base modifications regulate (4) mRNA degradation, (5) mRNA stability, (6) RNA structure, and (7) mRNA translation efficiency.

The dynamic and reversible RNA base modifications are catalyzed by distinct enzymes like methyltransferases (writers), and removed by demethylases (erasers). These modifications are interpreted by a modification-specific binding proteins known as readers. Characterization of writers, readers, and erasers is further advancing our epitranscriptomic understanding of functional genomics. Similar to the epigenetic modifications of DNA bases (Kumar et al., 2018), mRNA base modifications provide another layer of information created by the writers/erasers and interpreted by the readers. Like the reversible nature of DNA base modifications (Wang et al., 2016), some of the mRNA base modifications are known to be reversed by their respective eraser. Although translation process is typically controlled by translation factors and certain non-coding RNAs (ncRNAs), base modifications play equally important role in mRNA metabolism and translation process. Thus, the mRNA base modifications create the epitranscriptomic regulatory machinery that is being elucidated in the animal as well as the plant systems. It is now apparent that mRNA is a dynamic and reversibly modifiable biomolecule (Figure 2) that play crucial roles in post-transcriptional regulation of gene expression (Zhao et al., 2017a).

Many of the mRNA base modifications involve attachment of a methyl (CH_3_) group at a particular position either on the base [e.g., *N*^6^-methyladenosine (m^6^A), *N*^1^-methyladenosine (m^1^A), 5-methylcytidine (m^5^C), 3-methylcytidine (m^3^C), *N*^7^-methylguanosine (m^7^G), and 1-methylguanosine (m^1^G)], ribose sugar (e.g., 2′-*O*-methyladenosine), or on both base and sugar [e.g., *N*^6^,2′-*O*-dimethyladenosine (m^6^Am)] (Dominissini et al., 2012; Linder et al., 2015; Dominissini et al., 2016; Molinie et al., 2016; Mauer et al., 2017). Thus, methylation of bases at different position has distinct impact on RNA biology by affecting folding, stability, cellular localization, and/or interaction with other RNAs/proteins (Wu et al., 2016). The m^6^A is one of the most common, reversible epitranscriptomic marks, functionally pertinent in both animal and plant mRNAs (Batista et al., 2014; Shen et al., 2016). Moreover, writers, readers, and erasers for m^6^A are known in animals as well as plants (Bokar et al., 1997; Zhong et al., 2008; Liu et al., 2014; Du et al., 2016; Liu and Pan, 2016; Patil et al., 2016; Roundtree and He, 2016; Martinez-Perez et al., 2017; Zhao et al., 2017b; Arribas-Hernandez et al., 2018; Scutenaire et al., 2018; Wei et al., 2018; Liang et al., 2020). The m^6^A destabilizes *A* = *U* pairing due to altered energetics/steric hindrance; however, the donor and acceptor in the hydrogen bond remain the same (Roost et al., 2015).

On the other hand, CH_3_ of m^1^A in RNA provides a positive charge (which interacts with negatively charged phosphate in the backbone) and it bulges out of the Watson–Crick hydrogen bond resulting in a strong electrostatic interaction (Helm, 2006). Moreover, 2′-*O*-methylation confers hydrophobicity, which protects the RNA from nucleolytic attack and stabilizes RNA coiling (Kumar et al., 2014). Thus, structure and functions of dynamic RNA modifications during the developmental process and environmental stress, and their effects on gene expression have emerged as a new branch of functional genomics known as ‘epitranscriptomics.’

To decipher the biological functions of a modified RNA base, it is vital to identify the writer/reader/eraser that modulates the modification. However, high-throughput detection methods for many of these modifications are still lacking. The recent advances in high-throughput next-generation sequencing (NGS) together with the novel chemogenetic RNA-labelng techniques have provided unprecedented opportunities to understand the RNA structure and functions. Such advances provide a better understanding of the presence and dynamics of base modifications like m^6^A (Zhao et al., 2017b), m^5^C (Cui Q. et al., 2017; David et al., 2017; Fang et al., 2020), 5-hydroxymethylcytidine (hm^5^C) (Huber et al., 2015; Delatte et al., 2016; Zhang et al., 2016), and m^1^A (Dominissini et al., 2016; Li et al., 2016a; Shen et al., 2016; Xiong et al., 2018) in RNAs. Base modifications, such as addition of 5′ cap (e.g., *N*^7^-methylguanosine, m^7^G-cap), and RNA editing are vital for mRNA stability (Kiledjian, 2018), translation (Topisirovic et al., 2011; Holstein et al., 2016) and functional diversity (Peng et al., 2018). More importantly, NAD^+^ has been reported to be a new/alternative RNA cap in diverse organisms including bacteria, yeast, human (Cahová et al., 2015; Jiao et al., 2017; Walters et al., 2017; Frindert et al., 2018), and plant (Wang et al., 2019). Thousands of transcripts for the protein-coding genes from nuclear and mitochondrial genomes in Arabidopsis were observed to contain NAD^+^ cap (Wang et al., 2019). These clearly indicate that NAD^+^ cap is one of the evolutionarily conserved caps that affects mRNA metabolic processes. A comprehensive understanding of the distribution, function, and regulation of RNA base modification will further increase the available knowledge on epitranscriptomic regulation of gene expression.

Epitranscriptomic base modifications have become an interesting topic of research and review, particularly in the animal system (Meyer et al., 2012; Carlile et al., 2014; Fu Y. et al., 2014; Dominissini et al., 2016; Peer et al., 2017; Angelova et al., 2018; Arribas-Hernandez et al., 2018; Khoddami et al., 2019; Leonardi et al., 2020). Now, the epitranscriptomic modifications in plants like Arabidopsis (Luo et al., 2014; Wan et al., 2015; Shen et al., 2016; Zuber et al., 2016; Cui Q. et al., 2017; David et al., 2017; Duan et al., 2017), rice (Li Y. et al., 2014), maize (Luo et al., 2019; Miao et al., 2019), and tomato (Zhou et al., 2019) are also being studied. However, our knowledge of plant epitranscriptomic modifications, except for the 5′-cap and poly-A-tail, is limited to uridylation (de Almeida et al., 2018), m^6^A (Li et al., 2018), and m^5^C (Cui X. et al., 2017; David et al., 2017). Other types of modifications can also be expected to occur in plant mRNAs but their existence/detection and roles/functions remain to be explored. Considering the crucial and dynamic roles of epitranscriptomic modifications in many biological processes like embryo development, leaf morphogenesis, root development, floral transition, fruit ripening, and stress tolerance, the importance and future perspectives of epitranscriptomic research in plants are being discussed (Hu et al., 2019; Shen et al., 2019; Liang et al., 2020). The present review focuses on recent developments in base modifications in RNAs, particularly m^6^A and m^5^C in plant mRNAs, their biochemical properties, and functions. Moreover, the review discusses technological advances in high-throughput detection methods to elucidate epitranscriptomic modifications, as well as the technological limitations. Further advances in the next-generation detection techniques and functional analysis of RNA base modifications might facilitate epitranscriptomic manipulation of the traits of interest.

## Biochemistry of Adenosine Methylation in mRNA

Methylation of adenosine (A) at *N*^6^ position [in both *syn*- (energetically favored) and *anti*-conformation] results in the formation of m^6^A (Zou et al., 2016). The methyltransferase-like 14 (METTL14) complex and Wilm’s tumor-associated protein (WTAP) work in cooperation with METTL3, and cofactors KIAA1429, RBM15/RBM15B which constitute a functional methyltransferase to create m^6^A in mammalian mRNAs at a consensus sequence of R−−m^6^A−C−H (where R = A/G, and H = A/C/U) (Patil et al., 2016). Emerging evidence suggests that VIRMA/KIAA1429 recruits the catalytic core (METTL3/WTAP/METTL14) for a sequence-specific methylation of A to m^6^A (Yue et al., 2018). Recent studies suggest that ZC3H13 is another component of the m^6^A writer-complex, and it regulates the methylation of A (Knuckles et al., 2018; Wen et al., 2018). Moreover, m^6^A mark gets erased by the enzymes like fat mass and obesity-associated protein (FTO) and alkylation repair homolog protein 5 (ALKBH5), which convert it back to A (Jia et al., 2011). FTO oxidatively removes m^6^A through *N*^6^-hydroxymethyladenosine (hm^6^A) and *N*^6^-formyladenosine (f^6^A) intermediates (Fu Y. et al., 2014). Thus, m^6^A is a reversible epitranscriptomic modification, which functions to regulate gene expression.

### m^6^A Writer

An RNA methyltransferase complex is comprised of methyltransferase-like 3 (METTL3) (Bokar et al., 1997), METTL14 (Liu et al., 2014), KIAA1429/VIRMA (Schwartz et al., 2014b; Yue et al., 2018), HAKAI (Ruzicka et al., 2017), RNA binding motif protein 15 (RBM15) (Patil et al., 2016), Wilm’s tumor 1-associating protein (WTAP) (Ping et al., 2014), and a zinc finger CCCH domain-containing protein 13 (ZC3H13) (Frye et al., 2018; Wen et al., 2018; Yang et al., 2018). It is involved in methylation/modification of adenosine to m^6^A in mammals. While METTL3 is known to methylate single-stranded RNAs (ssRNAs) in a sequence-specific (RRACH) manner, METTL16 methylates structured RNAs having a nonamer sequence (UAC**A**GAGAA; the targeted adenosine for methylation is marked with bold face) (Pendleton et al., 2017). Thus, METTL16 is another m^6^A-specific methyltransferase which targets U6 snRNA and human *MAT2A* mRNA encoding for S-adenosylmethionine (SAM) synthetase (Pendleton et al., 2017). Interestingly, SAM is the methyl group donor for methylation of DNA, RNA, and proteins.

In Arabidopsis, the m^6^A writer complex is composed of adenosine methyltransferase (MTA) (METTL3 ortholog), its homolog MTB (METTL14 ortholog), FKBP12 interacting protein 37 (FIP37) (WTAP ortholog), VIRLIZER/KIAA1229 (VIR), and HAKAI (Ruzicka et al., 2017) (Table 1). Although the components of plant writer complex were observed to be distributed in the nucleoplasm, but FIP37 and VIR do not affect alternative splicing of transcripts (Shen et al., 2016; Ruzicka et al., 2017). While WTAP interacts with METLL3, METTL14, VIRMA, and HAKAI in mammals (Yue et al., 2018), Arabidopsis FIP37 (a WTAP ortholog in mammals) interacts directly with MTA only (Ruzicka et al., 2017). This clearly indicates that the mechanism of adenine methylation (m^6^A) is conserved among the eukaryotes; however, some unique features of m^6^A modification might have been evolved in plants. Most of the constituents of m^6^A writer complex, excluding HAKAI, are needed for the embryonic development. Moreover, m^6^A plays diverse roles in various other developmental processes in plants. Hence even after conserved m^6^A modification machinery in eukaryotes, it appears that individual members of m^6^A writer complex has achieved functional divergence in plants.

**TABLE 1:** Modified RNA bases, their modulators, and interpreters.

RNA base modification	Enzymes/proteins						References
	Writer		Eraser		Reader		
	Animal	Plant	Animal	Plant	Animal	Plant	
Pseudouridine (Ψ) 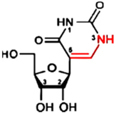	PUS1, PUS2, PUS3, PUS4, PUS6, PUS7, PUS9, PUS13 DKC1, BoxH/ACA	?	?	?	?	?	Carlile et al., 2014; Lovejoy et al., 2014; Spenkuch et al., 2014; Rintala-Dempsey and Kothe, 2017; Adachi et al., 2019; Khonsari and Klassen, 2020
*N*^6^-methyladenosine (m6A) 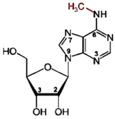	METTL3, METTL14 METTL16 WTAP RBM15B VIRMA ZC3H13 HAKAI Spenito	MTA, MTB FIP37 VIR HAKAI	ALKBH5 FTO	ALKBH9B ALKBH10B SIALKDH2	YTHDC1 YTHDC2 YTHDF1 YTHDF2 YTHDF3 eIF3 HNRNPC HNRNPA2B1 SRSF2	ECT2 ECT3 ECT4 COSF30L	Zhang et al., 2010; Zheng et al., 2013; Liu et al., 2014; Wang et al., 2015; Du et al., 2016; Patil et al., 2016; Martinez-Perez et al., 2017; Arribas-Hernandez et al., 2018; Pendleton et al., 2017; Scutenaire et al., 2018; Wei et al., 2018
*N*^1^-methyladenosine (m1A) 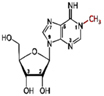	TRMT61B, TRMT10C, and the complex of TRMT6, TRMT61A	?	ALKBH1 ALKBH3	?	?	?	Chujo and Suzuki, 2012; Dominissini et al., 2016; Li et al., 2016a; Liu et al., 2016
*N*^6^,2′-*O*-dimethyladenosine (m6Am) 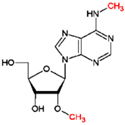	CMTR1 CMTR2 PCIF	?	FTO	?	?	?	Belanger et al., 2010; Jia et al., 2011; Werner et al., 2011; Mauer et al., 2017; Boulias et al., 2019; Sun et al., 2019
5-methylcytidine (m5C) 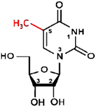	NSUN2 DNMT2	TRM4B	?	?	ALYREF YBX1	?	Squires et al., 2012; Hussain et al., 2013; Cui X. et al., 2017; David et al., 2017; Yang et al., 2017; Yang Y. et al., 2019
5-hydroxymethylcytidine (hm5C) 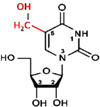	TET1, TET2, TET3	?	?	?	?	?	Fu L. et al., 2014; Huber et al., 2015; Delatte et al., 2016

*Modified RNA bases: Ψ, pseudouridine; 6-mA, N^6^-methyladenosine; 6-mAm, N^6^,2′-O-dimethyladenosine; 1-mA, N^1^-methyladenosine; m5C, 5-methylcytidine; hm5C, 5-hydroxymethylcytidine. ALKBH5, AlkB homolog 5; ALYREF, Aly/REF export factor; CMTR1, cap methyltransferase 1; DKC1, Dyskeratosis congenital protein 1; DNMT2, DNA methyltransferase 2; ECT2, Evolutionarily Conserved C Terminal region 2; eIF3, eukaryotic translation initiation factor 3; FIP37, FKBP12 Interacting Protein 37KD; FTO, fat mass and obesity-associated protein; HAKAI, a conserved E3 ubiquitin ligase in Arabidopsis; HNRNPA2B1, HNRNPC-Heterogeneous nuclear ribonucleoproteins A2/B1; KIAA1429, protein virilizer homolog; METTL3, methyltransferase-like 3; MTA, adenosine methyltransferase; MTB, closest homolog of MTA; NSUN2, NOL1/NOP2/Sun RNA methyltransferase family member 2; PUS1–PUS4, Pseudouridine synthase 1–4; RBM15, RNA-binding motif protein 15; SRSF2, serine/arginine-rich splicing factor 2; TET1–TET3, 10—11 translocation protein 1–3; TRM4B, tRNA-specific methyltransferase 4B; TRMT61B, tRNA-1-mA methyltransferase 61B; VIR, VIRLIZER/KIAA1229; WTAP, Wilms’ tumor 1 associated protein; YTHDF1–3, YTH domain family proteins 1–3; YTHDC1, YTH domain-containing protein 1; ZC3H13, CCCH-type zinc finger proteins. “?” indicates the unknown writer/eraser/reader.*

### m^6^A Reader

Methyladenosine (m^6^A) affects several mRNA metabolic processes in both nucleus and cytoplasm through the recruitment of m^6^A-binding protein (RBP), also known as m^6^A reader (Wang et al., 2015; Yue et al., 2015; Zhou et al., 2015; Xiao et al., 2016; Li A. et al., 2017; Yang et al., 2017; Zhao et al., 2017b; Scutenaire et al., 2018; Wei et al., 2018). Two important classes of m^6^A readers known so far include the YTH domain-containing protein (Zhang et al., 2010) and the heterogeneous nuclear ribonucleo-protein (HNRNP) (Alarcon et al., 2015a). Arabidopsis and rice genomes contain several genes (13 and 12, respectively) for the YTH homolog known as ‘evolutionarily conserved C-terminal region’ (ECT) (Li D. et al., 2014); however, their role as an m^6^A-reader has only recently been recognized (Arribas-Hernandez et al., 2018; Scutenaire et al., 2018; Wei et al., 2018). These genes exhibit distinct/diverse expression pattern in different organs at different developmental stages, and under different stress conditions. A plant-specific motif URUAW (R = G or A; W = U or A) was reported in the ECT2-binding sites, which is different from the YTH-binding motif observed in human (Xiao et al., 2016; Shen et al., 2019). The binding of ECT2 at m^6^A increases the stability of the transcript responsible for trichome morphogenesis/development in Arabidopsis. ECT2 functions with ECT3 and ECT4 to regulate leaf formation/morphogenesis (Arribas-Hernandez et al., 2018). Structural analysis of the m^6^A binding domain in yeast and mammalian YTH revealed that the it recognizes m^6^A in mRNA through a hydrophobic aromatic cage containing three conserved tryptophan residues (Luo and Tong, 2014; Theler et al., 2014; Xu et al., 2014). Mutation in the hydrophobic cage of ECT2 and ECT3 was reported to abolish the function of m^6^A recognition (Arribas-Hernandez et al., 2018; Wei et al., 2018; Scutenaire et al., 2018), which suggest that specific-binding of ECT to m^6^A is essential for their functional activity in leaf and trichome development. Occurrence of a number of YTH proteins in Arabidopsis and rice, having very high sequence similarity (Li D. et al., 2014), might help elucidating their roles in interpreting m^6^A epitranscriptome in plants by creating/using multiple knockout mutants.

### m^6^A Eraser

Since the formation of m^6^A is a reversible process, it is dynamically removed from the mRNA by two ALKBH family m^6^A demethylases namely ‘Fat mass and obesity-associated protein’ (FTO) (Jia et al., 2011) and α-ketoglutarate-dependent dioxygenase homolog 5 (ALKBH5) (Zheng et al., 2013) in mammals. In Arabidopsis (13) and rice (9) a number of ALKBH family proteins have been reported (Mielecki et al., 2012; Liang et al., 2020). Phylogenetic analysis showed no orthologs of FTO to be present in plants (Liang et al., 2020). But, the existence of multiple copies of ALKBH5 orthologs [six orthologs (ALKBH9A/B/C and ALKBH10A/B/C) in Arabidopsis] suggests redundant functions of these proteins in m^6^A demethylation. They are differentially expressed in different tissues (Duan et al., 2017) with their diverse subcellular localization (Mielecki et al., 2012). This again suggests their role in functional divergence in m^6^A dynamics in plants (Burgess et al., 2016). ALKBH9B, ALKBH10B, and SLALKBH2 (Zhou et al., 2019) remove m^6^A from mRNA in Arabidopsis (Duan et al., 2017; Martinez-Perez et al., 2017). ALKBH10B removes m^6^A from mRNAs for several regulators, which enhances the stability of the transcripts and promotes floral transition. Thus, m^6^A promotes degradation of mRNAs for developmental regulators in Arabidopsis (Duan et al., 2017). This indicates that it might potentially be used as an epitranscriptomic mark for modulating flowering time in crop plants.

### Occurrence of m^6^A

Occurrence of m^6^A has been observed across the animals, plants, single-cell organisms (archaea, bacteria, and yeast), and viruses (Zhao et al., 2017a). Three independent studies showed consensus on adenosine methylation (m^6^A) motif RRACH in yeast, mammals, and plants (Dominissini et al., 2012; Schwartz et al., 2013; Luo et al., 2014). It has been detected in mRNAs of many plant species, including Arabidopsis, maize, wheat, oat, and rice (Zhong et al., 2008). In Arabidopsis, m^6^A content varies in different tissues, ranging from 0.4% in seeds to 1.5% in young seedlings (Zhong et al., 2008). Three independent studies reported m^6^A mapping in different ecotypes and tissues of Arabidopsis (Luo et al., 2014; Wan et al., 2015; Shen et al., 2016). m^6^A was reported to be widely distributed in >5,000 transcripts, and accumulated near the start and the stop codons, as well as in the 3′ UTR (Luo et al., 2014). However, occurrence of m^6^A near the start codon was not detected in the methylome of leaf, flower, and root of Arabidopsis (Wan et al., 2015), probably because of the dynamic nature of the modified m^6^A. Differentially methylated mRNAs were observed in leaf, flower, and root of Arabidopsis (Wan et al., 2015), indicating the role of m^6^A in tissue/organ differentiation. The m^6^A writers MTA/MTB, FIP37/VIRILIZER/HAKAI were reported to be involved in embryo and plant development (Shen et al., 2016; Ruzicka et al., 2017; Hu et al., 2019). YTH/ECT and ALKBH, reader and eraser, respectively, play important role in growth, development and flowering in Arabidopsis (Duan et al., 2017; Arribas-Hernandez et al., 2018; Scutenaire et al., 2018; Wei et al., 2018). Differential methylation of several transcripts in root, leaf, and flower of Arabidopsis (Wan et al., 2015), suggests that m^6^A dynamics of specific transcripts might be an integral part of tissue/organ differentiation in plants (Shen et al., 2019). Another recent work on epitranscriptomic profiling of salt-treated Arabidopsis leaf reported m^6^A enrichment in the transcripts for salt- and osmotic-stress responses (Anderson et al., 2018).

In addition to Arabidopsis, the enzymes associated with epitranscriptomic modifications have been reported in some of the agronomically important plants like *Nicotiana sylvestris*, maize, rice, and tomato. The methylases and demethylases have also been reported in plants, and they are evolutionarily conserved. Any change in their expression shows a significant alteration in the m^6^A content in polyadenylated transcriptome, and drastic physiological impacts. Analysis of m^6^A landscape in rice (Li D. et al., 2014) exhibited a similar pattern that was observed in Arabidopsis, which indicates a conserved m^6^A distribution in plants. Accumulating evidences also indicate that writers/readers and erasers play important roles in abiotic stress responses in plants (Hu et al., 2019). Zhang F. et al. (2019) identified a panicle-specific m^6^A motif UGWAMH (W = U/A; M = C/A; H = U/A/C) in rice. Despite the progress being made in understanding m^6^A landscape in crop plants, the writers, readers, erasers for m^6^A and its functions in plant growth, development, and survival under the stress are yet to be elucidated. However, the position, pattern, and motif of m^6^A suggest that the writers, readers, and erasers might be conserved across the kingdoms.

### Methylation at Other Positions in Adenosine

In addition to the m^6^A, the human epitranscriptome is known to contain other modified/methylated forms of adenosine like m^1^A and m^6^Am (Hauenschild et al., 2015; Molinie et al., 2016). Methylation at the *N*^1^ position of adenosine creates *N*^1^-methyladenosine (m^1^A), and it has been prevalent in rRNA and tRNA. However, the occurrence of m^1^A has also been reported in the human transcriptome (Li X. et al., 2017), which can be erased by ALKBH3 (Li et al., 2016b). The CH_3_ group at *N*^1^ position of m^1^A interferes with standard base pairing (Hauenschild et al., 2015), which affects mRNA folding around the transcription start site (TSS) and facilitates initiation of translation. Despite the progress in the detection of modified nucleosides, transcriptome-wide distributions of m^1^A in plants remain unknown. When adenosine is methylated at the C_2_ position of ribose sugar [by 2′-*O*-methyltransferase (CMTR: Cap methyltransferase) to form 2′-*O*-methyladenosine (Am) (Werner et al., 2011) and then it is methylated at the *N*^6^ position of adenosine [by an unidentified nucleo-cytoplasmic methyltransferase], it forms *N*^6^, 2′-*O*-dimethyladenosine (m^6^Am). The m^6^Am modification is exclusively distributed at the TSS (generally after the m^7^G cap) in certain mRNAs (Linder et al., 2015) at a frequency of 0.003% (Molinie et al., 2016). m^6^Am was reported to be mediated by phosphorylated CTD interacting factor 1 (PCIF1) which catalyzes methylation of m^6^A to m^6^Am at the 5′ end of mRNA (Sendinc et al., 2019). Although such epitranscriptomic modifications play important roles in mammals, they are remained to be identified/characterized in plant.

## Modification of Other Bases in mRNA

Besides the modifications of adenosine, epitranscriptome is known to contain methylation/modification at other bases, for example, m^5^C, hm^5^C, m^3^C, ac^4^C, m^1^G, m^7^G, 8-oxo-G, Uridylation, Pseudouridine (ψ), and Inosine (I), particulately in animal systems (reviewed by Shen et al., 2019; Boo and Kim, 2020). While the occurrence of some of the modified bases (e.g., m^5^C, hm^5^C, m^7^G, and ψ) have been confirmed (Huber et al., 2015; Vandivier et al., 2015; Burgess et al., 2016; Zuber et al., 2016; Cui Q. et al., 2017; Martinez-Perez et al., 2017; Malbec et al., 2019), presence of m^1^G has been predicted in Arabidopsis epitranscriptome. Many of these epitranscriptomic modifications like m^3^C, m^7^G, 8-oxoG, and I play important roles in animals (Palladino et al., 2000; Torres et al., 2014; Arimbasseri et al., 2016; Xu L. et al., 2017; Malbec et al., 2019), but their existence/identification and functional characterization remains to be confirmed in plants.

### Cytosine Modifications in mRNA

Occurrence of methylcytidine (m^5^C) is common in tRNAs and rRNAs (Squires and Preiss, 2010), but it has also been identified in mRNAs and ncRNAs (Squires et al., 2012). Since m^5^C is less abundant (0.4% of total cytosine, compared to ∼1.5% of m^6^A in human transcripts), much less has been researched on its occurrence and functions (Squires et al., 2012; Ke et al., 2015). Detection of m^5^C in mRNAs of different plant species, including Arabidopsis, Medicago, rice, maize, and foxtail millet, has been reported (Cui Q. et al., 2017). Change in m^5^C level across the tissues in Arabidopsis, with a gradual increase during vegetative growth, suggest a dynamic change in m^5^C content during plant growth and development. More than one thousand m^5^C were detected on transcriptome-wide analysis of shoot, root, and siliques of Arabidopsis, but only a few dozen of them were commonly present among these tissues (David et al., 2017). m^5^C is generally accumulated in the coding sequence (CDS) of the mRNA in HACCR (where H = A, U or C; R = A or G) and CTYCTYC (Y = U or C) motifs in Arabidopsis (Cui Q. et al., 2017). A marginal increase in expression of TRM4B (an m^5^C writer) was observed under cold stress in Arabidopsis, but it showed decreased expression under heat stress. However, the expression level of TRM4B was not altered in rice under abiotic stresses (Zou et al., 2016). TRM4B has been further characterized in plants (David et al., 2017; Cui Q. et al., 2017), and m^5^C was observed to be required for root development and oxidative stress responses (David et al., 2017). TRM4B loss-of-function mutants of Arabidopsis exhibited down-regulated expression of short hypocotyl 2 (SHY2) and indoleacetic acid-induced protein 16 (IAA16) genes involved in root development. Stability of the transcripts of such genes was observed to be positively correlated with the m^5^C modification/content (Cui Q. et al., 2017).

### Writer, Reader, and Eraser of m^5^C

Formation of m^5^C in human mRNA is catalyzed by methyltransferases such as DNMT2 and NSUN2 (Squires et al., 2012; Bohnsack et al., 2019). NSUN6, a Type II m^5^C site-specific methyltransferase, was reported to negatively correlate m^5^C methylation with translation efficiency (Liu et al., 2020). Recently, Selmi et al. (2021) mapped NSUN6-dependent m^5^C sites in human transcripts, which were located in protein coding RNAs at 3′-UTR within a consensus sequence (CTCCA) motif, and mark translation termination. Eight m^5^C methyltransferases are encoded by Arabidopsis genome, two of them are tRNA-specific methyltransferase 4A (TRM4A) and TRM4B (Chen et al., 2010; Cui Q. et al., 2017). While TRM4A is responsible for m^5^C in tRNA, TRM4B targets mRNA for the modification. A recent study demonstrated that an RRM motif-containing ALY protein binds to m^5^C-containing mRNAs in Arabidopsis (Pfaff et al., 2018). The *aly* mutants showed shorter primary roots, defective reproductive development including abnormal flowers and reduced seed production (Pfaff et al., 2018). Thus, m^5^C is another important epitranscriptomic mark that affects plant growth, development and adaptive responses in plants. Although m^5^C is reported to be further oxidized to hm^5^C by a family of Ten-eleven translocation (TET) enzymes (Huber et al., 2015; Delatte et al., 2016), varying hm^5^C content in different Arabidopsis tissues indicate that it is a dynamic epitranscriptomic mark in plants (Shen et al., 2019). Despite the progress in detecting/distribution of hm^5^C, its oxidation to m^5^C in mRNA is still not fully demonstrated. However, further research would be required to identify m^5^C readers/erasers, and elucidate the mechanisms/functions of m^5^C-mediated regulation of gene expression.

### Methylation at Other Positions in Cytosine

Cytosine can also be acetylated at the *N*^4^ position by an *N*-acetyltransferase (NAT10) to form ac^4^C. Such modification is commonly found in tRNA, rRNA, but it has also been observed in mRNA (Dong et al., 2016; Arango et al., 2018). ac^4^C was observed distributed in coding and non-coding RNAs in human, abundant near the TSS (Arango et al., 2018). The occurrence of ac^4^C increases mRNA half-life and promotes translation efficiency. NAT10 acts as the primary ac^4^C writer, and NAT10 knocking out reduces ac^4^C content in RNA. In yeast, orphan box C/D snoRNAs complex guides Kre33 (a yeast homolog of human NAT10) to the target sites for ac^4^C modification (Sharma et al., 2017). However, it is still not known whether ac^4^C is a reversible or not, as neither an ac^4^C reader nor its deacetylation process is known. Moreover, its occurrence in plant and role/function in gene regulation is not yet known.

### Modification of Other Bases in mRNA

Uridylation (addition of uridines at the 3′ without any template) of mRNA, targeted for degradation, has been reported in both mice and Arabidopsis (Shen and Goodman, 2004; Zhang et al., 2017). Uridylation of mRNAs in plants is catalyzed by UTP: RNA uridylytransferase1 (URT1) and terminal uridylyltransferase (TUTase) (Sement et al., 2013; Lim et al., 2014). Pseudouridine (Ψ), also known as the 5th base of RNA and the first modified RNA base (Davis and Allen, 1957), is a C−glycosidic rotational isomeric form of uridine (U), wherein U is attached to a ribose sugar through a carbon–carbon (instead of a nitrogen-carbon) glycosidic bond. Formation of Ψ in eukaryotes involves an RNA-dependent pseudouridine synthase (PUS) such as Cbf5 which uses a cofactor box (H/ACA ribonucleo-proteins) as a guide. Ψ formation may also occur through an RNA-independent PUS that does not require any cofactor (Carlile et al., 2014; Spenkuch et al., 2014). Ψ may further get methylated by EMG1 at the *N*^1^ position to generate 1-methylpseudouridine (m^1^Ψ) (Wurm et al., 2010). Although Ψ is mainly distributed around the CDS and the 3′ UTR of the gene (Carlile et al., 2014; Li et al., 2015), its occurrence is yet to be mapped in plant mRNAs.

Oxidation of RNA bases due to excessive reactive oxygen species (ROS, e.g., superoxide, hydroxyl radicals, and hydrogen peroxide) generates different oxidized RNA bases like 8-oxoG, 8-oxo-7,8-dihydroadenosine, 5-hydroxycytidine, cytosine glycol, and 5-hydroxyuridine (Yan and Zaher, 2019). 8-Oxo-7,8-dihydroguanosine (8-oxoG, an oxidized form of guanine base) is one of the most abundant variants of guanosine found in mammalian cells associated with neurodegenerative diseases (Nunomura et al., 2017). This determines the fate of mRNA, including stability and translation (Yan et al., 2019). AU-rich element RBP 1 (AUF1) and Y-box binding protein 1 (YBX1) preferentially bind to 8-oxoG to trigger rapid degradation of 8-oxoG–containing mRNAs (Ishii et al., 2015). Recently poly(C)-binding protein 1 (PCBP1) was identified as an 8-oxoG reader protein. However, the binding of PCBP1 requires two 8-oxoGs located nearby in the RNA, and this is associated with cellular apoptosis under oxidative stress (Ishii et al., 2018). Reversal of 8-oxoG to a normal guanosine base, as observed in the case of many other RNA base modifications, is not yet known. Moreover, the occurrence of such modified RNA base(s) in plant can be expected, particularly under environmental stresses when ROS production increases significantly, but their existence has not yet been reported.

## Effect of Modified Base on mRNA Metabolism

Modified bases influence mRNA metabolism, including splicing, export, translation, and degradation of the transcript. Many of the functions of m^6^A in mRNA metabolism in animal system are well-known (Wang et al., 2014; Liu et al., 2015; Haussmann et al., 2016; Shi et al., 2017; Kasowitz et al., 2018). However, only some of the functions of m^6^A and its readers like ECT2 are known in plants, including the regulation of 3′ UTR processing and improving mRNA stability (Wei et al., 2018). Moreover, some other functions of the core components of the methyltransferase complex (MTA, MTB, and FIP37) in plant development and survival under abiotic stresses were deciphered by mutation/knock-out studies in Arabidopsis (Tzafrir et al., 2003; Vespa et al., 2004; Zhong et al., 2008; Bodi et al., 2012). The function of another modified adenosine base, m^6^Am (a close homolog of m^6^A), is yet poorly understood, but it has been reported to improve translation efficiency and mRNA stability in mice by protecting the mRNA from decapping enzymes like DCP2 (Mauer et al., 2017).

m^5^C facilitates binding of ALYREF (an mRNA export adaptor) and removal of NSUN2 (an m^5^C writer), which disrupt mRNA transport from the nucleus (Yang et al., 2017). In Arabidopsis, a reduced ribosomal occupancy was observed in the m^5^C–marked mRNAs, indicating interfering role of m^5^C in binding of translational machinery (Cui Q. et al., 2017). A decreased m^5^C content accelerates mRNA decay, which indicates that it is another important epitranscriptomic mark affecting mRNA stability and translation efficiency in plants (Cui Q. et al., 2017).

Other modified bases, such as ψ, have been depicted to be involved in splicing and undisrupted translation of mRNA in yeast and mammals (Carlile et al., 2014; Karijolich et al., 2015). URT1-dependent uridylation and poly-A binding protein (PABP) in plants was reported to prevent excessive deadenylation, and thus, protects mRNA from degradation in Arabidopsis (Zuber et al., 2016). The occurrence of 8-oxoG considerably inhibits the efficiency of peptide bond formation, which restricts translation and triggers mRNA degradation (Boo and Kim, 2020). Transcription factors (TFs; e.g., ZFP217-dependent METTL3 and HIF-dependent ALKBH5), and miRNAs (e.g., miRNA responsible for RNA-dependent METTL3 activity) may also trigger the expression of writers and erasers of modified bases, demonstrating the feedback activation. This suggests a complex interplay between the modified bases and regulatory pathways. Thus, the stimuli and signaling/regulatory processes that fine-tune the transcription and translation processes of a gene might also affect the activity of writers, readers, and erasers through various RNA modifications. The same signaling pathway may also activate or inactivate the synthesis of readers and erasers through post-translational modifications.

### Role of Modified Base on mRNA Translation

Translation process is regulated by the binding of ribosome and initiation factor activities, including phosphorylation of the ‘eukaryotic initiation factor 2’ (eIF2) (Pavitt, 2018). Translation efficiency was reported to be moderately increased in the METTL3-knockout mutants of mouse embryonic stem cells and embryoid bodies, which suggest a negative regulatory role of m^6^A on translation efficacy (Liu et al., 2015). However, the binding of YTHDF1 (a cytoplasmic m^6^A reader) cooperates with ribosomes and initiation factors to increase translation efficiency (Wang et al., 2015). Recent studies demonstrate that m^6^A promotes translation efficacy of mRNAs (Li A. et al., 2017; Shi et al., 2017; Weng et al., 2018). Similarly, IGF2BPs (m^6^A-binding proteins) help reinforcing the stability and increase the translation efficiency of m^6^A-containg mRNAs (Huang et al., 2018). Studies also suggested that the presence of m^6^A in 5′ UTR of an mRNA promotes initiation of cap-independent translation (Meyer et al., 2015) and the IGF2BPs-mediated translation (Huang et al., 2018). It has also been reported that eIF3 directly binds to m^6^A−harboring 5′ UTR and engages the 43S ribosomal complex to begin the translation process, even in the absence of eIF4E (a cap-binding factor) (Meyer et al., 2015). The presence of m^6^A in the coding region of mRNA has been reported to disrupt tRNA boarding and elongation of the translation process *in vitro* (Choi et al., 2016). m^6^A has also been reported to negatively regulate the translation process by serving as a link between transcription and translation processes (Slobodin et al., 2017). All of these findings support the regulatory functions of m^6^A in mRNA translation.

Recent mapping studies indicate that m^1^A is abundant in the 5′ UTR of mRNA (Dominissini et al., 2016; Li X. et al., 2017), which is associated with higher translational efficiency; however, the underlying mechanism is yet to be discovered. In addition to this, the presence of m^6^Am creates hindrance in the binding of mRNA-decapping enzyme DCP2, which improves the stability of the transcript (Mauer et al., 2017). Moreover, m^6^Am also makes mRNA resistant to microRNA-mediated degradation (Mauer et al., 2017). Similarly, m^5^C has been reported to stabilize RNA secondary structure; hence, it influences translational fidelity (Helm, 2006; Squires and Preiss, 2010). While the presence of m^5^C at the first position in the CCC codon was reported to reduce translational product by ∼40% using bacterial whole-cell extract, its presence at the 2nd position of the codon was reported to suppress translation termination (Hoernes et al., 2016). In contrast, hm^5^C has been reported to activate translation in *Drosophila melanogaster* (Delatte et al., 2016). The effects of Ψ on translation efficiency depend on its position in a codon.

Although m^6^A has been known to promote translation efficiency in the animal system (Meyer et al., 2015; Wang et al., 2015; Slobodin et al., 2017), a little is known about its functions in plants where it works differently. In maize, m^6^A was found to be negatively correlated with translation efficiency; however, this depends on the location and content of m^6^A in the gene (Luo et al., 2019). Similarly, m^5^C was also reported to be associated with reduced efficiency of translation in Arabidopsis (Cui Q. et al., 2017). A recent study reports m^5^C to play important role in mRNA stability (Yang L. et al., 2019), which in turn improves translation efficiency. Thus, the role of different methylated bases in mRNA translation needs to be further explored to better understand the epitranscriptomic regulation of gene expression in plants.

### Role of Modified Base on mRNA Splicing, Export, and Decay

Transcripts with modified bases get easily exported, translated, and degraded, probably due to the binding of the reader at the modified base. Studies provide convincing evidence for the regulatory function of m^6^A on processing of pre-mRNA and pri-miRNA (Alarcon et al., 2015b). A family of nuclear hnRNPs, an m^6^A-binding protein accelerate processing of pri-miRNAs through interaction with DGCR8 (Alarcon et al., 2015a). An hnRNPA2B1 modulate alternative splicing of transcripts (Alarcon et al., 2015a). Moreover, hnRNPC plays an important role in the pre-mRNAs processing (Rajagopalan et al., 2012). Pfaff et al. (2018) reported that an RRM motif-containing ALY protein binds to m^5^C-containing mRNAs and helps in mRNA export in Arabidopsis. Reports suggest that controlling RNA modification regulates mRNA stability which ultimately fine tunes the gene expression. Research demonstrates that alternatively spliced mRNAs in animals retain more m^6^A sites and the binding sites for METTL3. Geula et al. (2015) reported that a METTL3-deficient mouse embryonic stem cell retains intron and shows exon skipping. Thus, m^6^A exerts its effect through binding of the reader proteins, particularly a family of proteins containing YTH domain (Xu et al., 2014). YTHDF2 (a well-established m^6^A reader) specifically binds to m^6^A−containing mRNA to deploy CCR4–NOT deadenylase complex (Du et al., 2016) for mRNA transport to the processing bodies (Wang et al., 2014), which promotes degradation of mRNA through translocation of the transcript (Sheth and Parker, 2003). This indicates a linkage between m^6^A and mRNA degradation. m^6^A modification and binding of readers also affect mRNA splicing and alternative polyadenylation (Xiao et al., 2016; Kasowitz et al., 2018). An alternative to 3′–5′ exoribonucleolytic cleavage on mRNA, endoribonucleolytic cleavage of the m^6^A-containing mRNAs is mediated by interaction among the YTHDF2, heat-responsive protein 12 (HRSP12), and P/MRP (an endoribonuclease RNase) complex (Park et al., 2019). Presence of the 8-oxoG in mRNA causes ribosome stalling followed by no-go decay (Ikeuchi et al., 2018). The roles of modified RNA base in regulation of mRNA stability/decay have recently been reviewed by Boo and Kim (2020).

### Effects of Methylated Base on Biological Processes

Complex cellular processes are intricately regulated by mRNA methylation. According to the cellular needs, mRNA export/localization is altered by RNA base methylation (Roundtree et al., 2017; Yang et al., 2017; Chen et al., 2019). The presence of m^6^A in transcripts of pluripotent TFs prompts transcriptomic flexibility in embryonic stem cells of mouse and human (Batista et al., 2014; Geula et al., 2015). Sequestration of METTL3 by ZFP217 indicates a complex interplay between epitranscriptome and TFs (Aguilo et al., 2015). The depletion of m^6^A from glioblastoma stem cells due to METTL3/14 knockdown was reported to promote self-regeneration and tumorigenesis (Cui X. et al., 2017). In Zebrafish (*Danio rerio*), m^6^A coordinates the elimination of maternal mRNAs with the help of Ythdf2 which is essential for maternal-to-zygotic transition (Cui X. et al., 2017). Heat-shock stress suppresses cap-dependent translation and induces adenine methylation (formation of m^6^A) at 5′ UTR of the transcripts (Meyer et al., 2015; Zhou et al., 2015). Although cells can discriminate between self (modified) and non-self (unmodified) RNAs, epitranscriptome plays an important role in immune responses also (Kariko et al., 2012; Hull and Bevilacqua, 2016). A study on the precursor cells of neurons revealed that m^5^C regulates differentiation and motility of neural stem cells in mice and humans (Flores et al., 2017). Mutants for FIP37 displayed about 85% reduction in m^6^A content and a massive proliferation of apical meristem in the shoot (Shen et al., 2016). Loss of m^6^A in FIP37 mutants of Arabidopsis was reported to be a key regulator of transcripts like WUSCHEL and SHOOTMERISTEMLESS, which results in the accumulation of transcripts due to their decreased decay (Shen et al., 2016).

Advances in epitranscriptomics have revealed several potential biological roles of post-transcriptional mRNA modifications (Zhao et al., 2017a). Reports demonstrate that methylated transcripts have shorter 3′ UTRs and lesser stability than its unmethylated counterpart (Molinie et al., 2016). Thus, methylation of mRNA base, and synthesis/binding of TFs/regulatory proteins get synchronized in response to the development processes and environmental stimuli (Zhao et al., 2017b). In mouse brain, the m^6^A level was reported to increase throughout the lifespan (Meyer et al., 2012). Studies have shown the role of m^6^A accumulation in learning and memory in mouse mediated by Ythdf1 binding in response to stimuli (Shi et al., 2018). Moreover, a recent study suggests the stress-mediated regulation of m^6^A accumulation in patients with depression, indicating that the dysregulation of m^6^A is associated with the development of mental disorders (Engel et al., 2017). An impaired build-up of m^6^A disrupts sex determination in *Drosophila*, and it causes embryonic-lethality in plants (Haussmann et al., 2016). Moreover, reduced accumulation of m^6^A inhibits the differentiation of embryonic stem cells in mammals (Batista et al., 2014; Geula et al., 2015). Some of the studies also suggest that the presence of m^6^A in mRNA plays a crucial role in spermatogenesis in mice (*Mus musculus* L.) (Hsu et al., 2017; Xu K. et al., 2017).

In Arabidopsis, the deficiency of mRNA adenosine methylase enzyme (a homolog of METTL3) has serious effects on plant growth and development (Bodi et al., 2012). Mutation studies on m^6^A methyltransferase core components (MTA, MTB, and FIP37) in Arabidopsis suggest that m^6^A is essential for the survival of the plant (Zhong et al., 2008). Arabidopsis mutants for FIP37 displayed an 85% reduction in m^6^A content and massive proliferation of apical meristem in the shoot (Shen et al., 2016). Knockdown of *MTB* in Arabidopsis was reported to cause a considerable reduction in height of the plant, while hypomorphic *vir* allele produced defective roots and the *VIR* null mutants were observed to be embryo-lethal (Ruzicka et al., 2017). A distinct pattern of m^6^A accumulation was observed in different organs of *Arabidopsis*, which suggests that m^6^A plays a role in organogenesis and it has tissue-specific functions (Wan et al., 2015). The content of m^6^A in Arabidopsis transcripts is controlled by 13 different ALKBHs (Mielecki et al., 2012) which indicate dynamic expression and diverse subcellular localization of ALKBH in plant. The *alkbh10b* mutants of Arabidopsis showed elevated m^6^A content in >1,000 transcripts and delayed floral transition, indicating that it mediates demethylation (removal of m^6^A) of regulatory transcripts (Duan et al., 2017). A wild-type *ALKBH10B* could restore the *alkbh10* mutant phenotype, suggesting that m^6^A is an important regulator of flowering time in plants. The regulatory function of ECTs in leaf morphogenesis and trichome development has recently been demonstrated (Arribas-Hernandez et al., 2018; Scutenaire et al., 2018; Wei et al., 2018). ECT2 binds at m^6^A sites in the trichome development-related genes and improves the mRNA stability. Transcripts of the genes in *ect2* mutant get degraded at an accelerated rate and affect trichome branching (Wei et al., 2018), which suggests that m^6^A mediates trichome and leaf development by the recruitment of reader proteins.

A large number of differentially methylated transcripts were observed in leaf, flower, and root of Arabidopsis, while >14,000 transcripts were found to contain m^6^A in rice leaf (Wan et al., 2015). Findings suggest that m^6^A might be involved in tissue-differentiation in plants. *OsMTA2* and *OsFIP* were identified to be the important components of RNA m^6^A methyltransferase complex, and m^6^A is involved in the regulation of sporogenesis, particularly male gametogenesis in rice (Zhang F. et al., 2019). A loss-of-function mutation in *OsFIP* resulted in the early degeneration of microspores, irregular meiosis in prophase I. Tomato *slalkbh2* mutants showed delayed fruit ripening phenotypes and increased m^6^A content compared with the wild-type plants (Zhou et al., 2019). SlALKBH2 is involved in the demethylation of the SlDML2 mRNA and regulates its degradation. SlDML2 encodes a DNA demethylase that regulates the expression of SlALKBH2 through DNA (5-mC) methylation. This suggests a novel mechanism of gene regulation connecting epigenetics (DNA methylation, 5-mC) and epitranscriptomics (mRNA modification, m^6^A) (Zhou et al., 2019). Loss of function mutation in Arabidopsis for TRM4B (an m^5^C writer) resulted in defective root phenotype because of the decreased content of m^5^C in the genes involved in root development (Cui Q. et al., 2017).

mRNA base modifications (m^6^A and m^5^C) are sensitive to environmental changes in plants. The m^5^C content was reported to decrease under drought and heat stress in Arabidopsis (Cui Q. et al., 2017). Similarly, m^6^A content was reported to decrease in drought stress (Zhou et al., 2019), suggesting the epitranscriptomic regulation of stress responses in plants. The findings indicate that m^6^A and m^5^C play important roles in post-transcriptional regulation of gene expression in plants. Several studies on the writers, readers, and erasers in plants demonstrate that mRNA modification is an important molecular mechanism for regulating plant development and environmental responses (Shen et al., 2016; Cui Q. et al., 2017; Duan et al., 2017; Martinez-Perez et al., 2017; Scutenaire et al., 2018). More importantly, most of the above-mentioned functions result due to silencing/over-expressing of gene or due to the combined action of reader/eraser but not only due to the removal/accumulation of any RNA base modification. Thus, the authors agree with the limitations of the studies/reports, and realize the importance of the factor(s) involved. Moreover, the mechanism for synchronized response of writers, readers, and erasers to internal/external stimuli is still elusive. Despite the progress in understanding the functions of m^6^A and m^5^C in plants, the mode of action of their writers, readers, and erasers are yet to be discovered.

## Detection of Modified Base in RNA

Post-transcriptional modifications in RNA bases have been reported to play essential roles in various functional RNAs. These modifications alter the structure, processing, and functions of RNAs. A comprehensive understanding of the biochemical modifications in mRNA bases and the changes in accompanying non-covalent interactions is required to gain insights into the functional diversity. m^6^A is the most abundant modified mRNA base and the first epitranscriptomic modification mapped (Dominissini et al., 2012; Meyer et al., 2012). Although marvelous progress has been made in understanding the modified mRNA bases (Liang et al., 2020), in-depth insights into the dynamics, structure, and functions of such epitranscriptomic modification in this fascinating messenger biomolecule are essential. Detection of the modified RNA base helps understanding its dynamics and biological functions. Modern high-throughput technologies together with the conventional methods (Table 2) are expected to advance the field of epitranscriptomics by generating data and discoveries. However, most of the current methods of detecting modified base are specific for a particular modification but recently Khoddami et al. (2019) reported a method (RNA bisulfite sequencing, RBS-seq) for transcriptome-wide detection of multiple base modifications (m^5^C, Ψ, and m^1^A) simultaneously at single-base resolution. In this section, we present an overview of the technological advancements in the detection methods, their applications, and their limitations.

**TABLE 2:** Techniques for detection of modified RNA base.

Method/technique	Base modification	Detection principle	References
Thin layer chromatography (TLC*), SCARLET	m^6^A, m^5^C	Difference in the net charge, polarity, and hydrophobicity. Radioactive (^32^P) labeling increases sensitivity of the SCARLET technique.	Grosjean et al., 2004; Barciszewska et al., 2007; Liu et al., 2013
HPLC, LC-MS/MS*, Dot-blot*.	m^6^A, m^1^A, m^5^C, hm^5^C	The RNA is digested into mononucleotides and detected on HPLC using UV light or mass spectrometry. In case of LC-MS/MS, modified base is quantified using the nucleoside-to-base ion mass transition. In dot-blot (a semiquantitative method), modified base-specific (e.g., anti-m^5^C) antibody is used to detect the modified base.	Jia et al., 2011; Kellner et al., 2014; Huber et al., 2015; Li et al., 2016a; Shen et al., 2016; Thuring et al., 2016; Cui X. et al., 2017; Limbach and Paulines, 2017
Single-molecule real-time (SMRT) technology.	m^6^A, m^1^A	The modified adenine (6-mA) can be discriminated from the unmodified adenine (A).	Vilfan et al., 2013; Dominissini et al., 2016
Chemical pretreatment approach, ICE-Seq	Inosine (I)	Acrylonitrile treatment causes inosine-specific cyanoethylation leading to the truncation of reverse transcription, allowing inosine (I) sites to be detected by subsequent RNA-sequencing.	Sakurai et al., 2014; Suzuki et al., 2015
Modification-specific RT signature technique	Inosine	The modified nucleotide leaves specific signatures in the cDNA sequences, which cause either abortive primer extension and/or misincorporation at or around the modified site.	Levanon et al., 2004
	m^1^A	Modified nucleotide affects cDNA synthesis either due to its inability to base-pair with its regular partner or by slowing down the rate of cDNA synthesis due to its massive or highly hydrophobic structure.	Hauenschild et al., 2015; Li X. et al., 2017
Biological/chemical induction of modification-specific RT signature	Pseudouridine (ψ),	Pseudouridine reacts with carbodiimide (CMCT) and forms a stable adduct, while U-CMC adducts are removed by alkaline treatment. The resulting ψ-CMC generates RT-arrest, which is detectable in the sequencing profile.	Schaefer et al., 2009
	m^5^C	5-mC is RT silent, but it is insensitive to bisulfite deamination. Cytosine (C) residue is deaminated into Uracil due to bisulfite treatment. The presence of C is detected by sequencing, wherein it is replaced by uracil.	Edelheit et al., 2013
	*N*^6^, 2′-*O*-dimethyladenosine (m^6^Am)	Ribose 2′-*O*-methylation protects the 3′-adjacent phosphodiester bond from alkaline cleavage which is used to identify the 2′-*O*-methylation site in RNA.	Marchand et al., 2016
Antibody-based method, MeRIP-isolated by crosslinking immunoprecipitation-seq (MeRIP-iCIP), MeRIP-qPCR*, MeRIP-seq*	m^6^A, m^5^C, hm^5^C, m^1^A	Modification-specific (anti-6-mA) antibody used to immunoprecipitate short RNA fragments, followed by cDNA libraries preparation and sequencing.	Dominissini et al., 2012; Meyer et al., 2012; Chen et al., 2015; Cui et al., 2016; Delatte et al., 2016; Shen et al., 2016; Li X. et al., 2017
Modified bisulfite (BS-seq*) strategy	m^5^C	Bisulfite treatment converts unmodified cytosine (C) to uracil, but 5-mC remains unchanged. The presence of C is detected by sequencing, wherein it is replaced by uracil.	Schaefer et al., 2009; Squires et al., 2012; David et al., 2017
*N*-cyclohexyl-*N*′-β-methylcarbodiimide (CMC-seq)	Ψ	CMC specifically labels Ψ forming CMC-Ψ adducts which stop RT at one nucleotide 3′ to the labeled Ψ site, thereby allows base-resolution detection of Ψ.	Schwartz et al., 2014a
Antibody-free method, MAZTER-seq, m^6^A-REF-seq, DART-seq, m^6^A-label-seq, m^6^A-SEAL*	m^6^A	Endoribonuclease-based RNA digestion with m^6^A-sensitive RNase (*Maz*F) at unmethylated ACA motif followed by sequencing (MAZTER-seq). In m^6^A-SEAL-seq method, DTT-mediated thiol-addition and FTO-mediated oxidation of m^6^A to hm^6^A as chemical labeling is utilized.	Garcia-Campos et al., 2019; Meyer, 2019; Zhang Z. et al., 2019; Shu et al., 2020; Wang et al., 2020

*Method used for detection of modified base in RNA in plant.

### Thin-Layer Chromatography

Thin-layer chromatography (TLC) has been one of the conventional methods for revealing RNA nucleobase modification (Keith, 1995). Generally, a modified base differs from its unmodified counterpart in terms of the net charge, polarity, and/or hydrophobicity, which allow their separation through chromatography. TLC separation of bases can be performed in one-dimension (1D) or two-dimensions (2D) using microcrystalline cellulose as a stationary phase. Using this method 2D-TLC maps for several modified RNA bases have been prepared (Grosjean et al., 2004; Barciszewska et al., 2007; Zhong et al., 2008). The sensitivity of the method can be increased by using radioactive (^32^P) labeling [site-specific cleavage and radioactive labeling, ligation-assisted extraction, and thin layer chromatography (SCARLET)] to detect the modification within an individual transcript (Liu et al., 2013). However, the TLC-based method fails to provide information about the location/context of the modified base.

### High-Performance Liquid Chromatography and MS

The content of modified RNA nucleobases can also be determined in digested mononucleosides by using high/ultra-performance liquid chromatography (UPLC) followed by mass spectrometry (MS) (Thuring et al., 2016). The method has been extensively used earlier for the detection and quantification of modified RNA bases. MS coupled to a nano-chromatography system reduces the amount (to picomole) of the sample required. Detection of a modified RNA base through ‘matrix-assisted laser desorption/ionization−time-of-flight’ (MALDI-TOF) is still being optimized (Schwartz and Motorin, 2017). Detection of the modified RNA bases can also be performed using methods like dot-blot and LC-MS/MS (Jia et al., 2011; Delatte et al., 2016; Shen et al., 2016; Cui Q. et al., 2017). Ross et al. (2016) reported LC-MS/MS based, sequence specific detection of modified nucleosides in tRNAs from bacteria and human.

### Reverse Transcription-Based Techniques

Reverse transcription (RT)-based techniques use the primer-extension method to reveal the modified RNA bases. The presence of the modified base in mRNA interrupts/inhibits primer extension, which facilitates its context-specific positioning. However, comparative sequence analysis with unmodified RNA transcripts is necessary to eliminate structural RT-stops. The advantage of the RT-based technique includes its applicability and sensitivity to a complex mixture of mRNAs, but it requires pure and concentrated mRNA molecules. Li X. et al. (2017) used a technique named m^1^A-MAP to detect the modified base at single-nucleotide resolution to profile m^1^A in the human transcriptome. Since m^1^A causes truncation and/or misincorporation during cDNA synthesis from the transcript (Hauenschild et al., 2015), a more precise method in detecting the position of m^1^A at single-base resolution. Nevertheless, inosine cannot be detected directly by the RT-based technique, as it can base-pair with cytosine. However, inosine-specific cyanoethylation treatment, using acrylonitrile, converts inosine into *N*^1^-cyanoethylinosine (ce^1^I) which disable base pairing of inosine with cytosine. This allows inosine sites to be detected by subsequent ‘inosine chemical erasing’ (ICE)-sequencing (ICE-Seq) in mRNA (Suzuki et al., 2015).

### NGS Technologies-Based Method for Detection of RNA Modification

**FIGURE 3:**
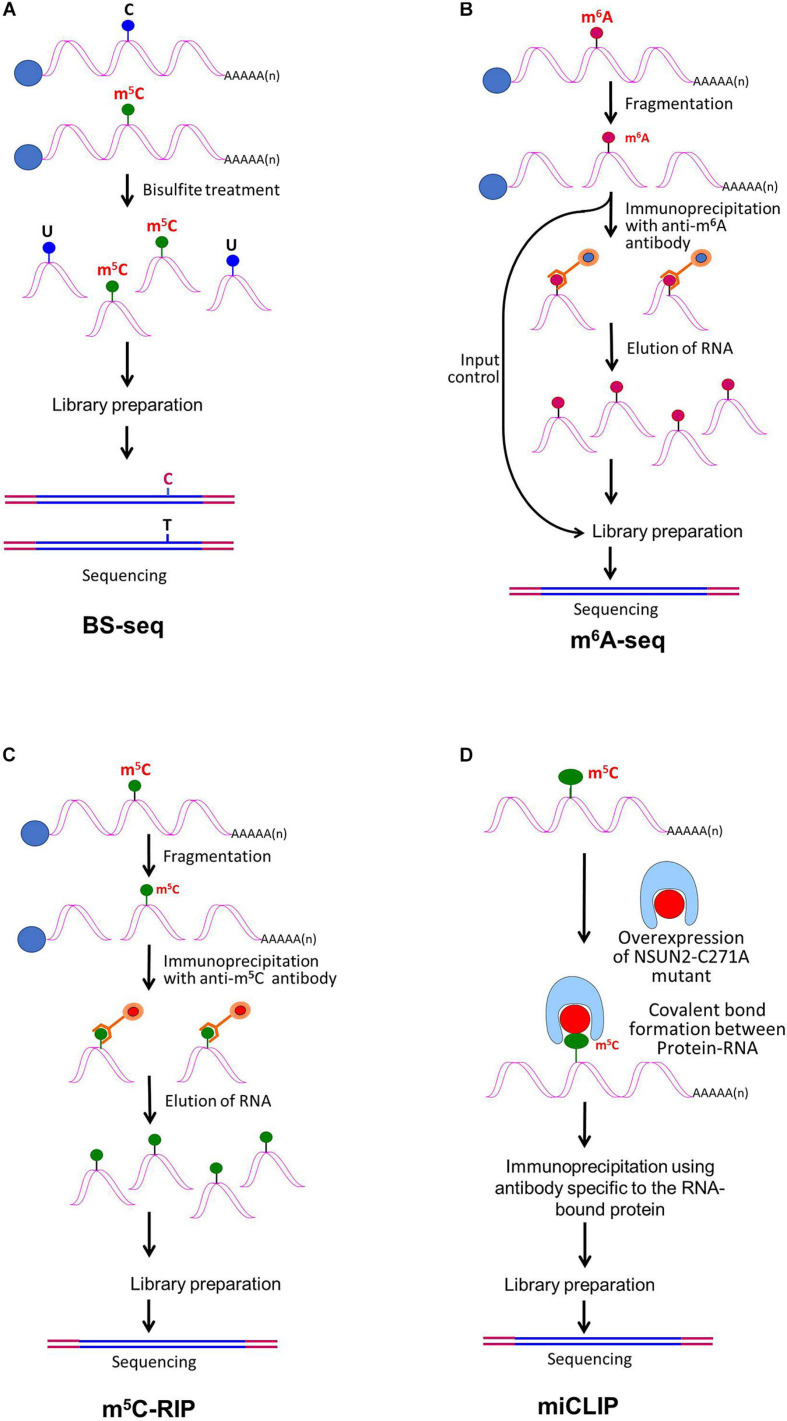
Detection of modified bases in mRNA. **(A)** Bisulfite sequencing (BS-seq) for the detection of 5-methylcytosine (m^5^C). Purified mRNA is fragmented into small (100–200 nt) fragments, and subjected to bisulfite treatment. Bisulfite treatment causes converts cytosine (C) to uracil (U), but m^5^C remains unchanged. Presence of C is detected by sequencing, wherein it is replaced by T. **(B)** Purified mRNAs are fragmented into 100–200 nt, followed by immunoprecipitation using anti-m^6^A antibody to enrich the sample with fragments containing the modified base, library preparation, and high-throughput deep-sequencing for detection of m^6^A. **(C)** Purified mRNAs are fragmented followed by immunoprecipitation using anti-m^5^C antibody of the fragments containing the modified base, library preparation, and sequencing. **(D)** m^5^C individual-nucleotide-resolution crosslinking and immunoprecipitation (m5C-miCLIP) exploites catalytic activity of cysteine-to-alanine mutation (C271A) mutant of NSUN2 (methyltransferase) which inhibits release of the enzyme from the protein–RNA complex making stable covalent bond between NSun2 and its RNA targets. Antibody specific to the RNA bound protein is used for immunoprecipitation, followed by library preparation and sequencing. This allows detection of low-abundance methylated RNAs without the need of deep sequencing.

The advances in next-generation high-throughput sequencing technologies for the detection of RNA base modifications have considerably improved the epitranscriptomic studies (Li et al., 2016b). Currently, the sequencing technologies employ an amplification step to generate clusters, which provides exceptionally high sequencing output with <0.1% error. However, the read length remains shorter (500–600 nt) in most of the cases. The BS-seq method combines bisulfite conversion followed by NGS to map m^5^C, which has been successfully used for epitranscriptomic analyses in several animals and plants (Squires et al., 2012; David et al., 2017). Although the BS-seq method precisely identifies the site of m^5^C at single-base resolution (Figure 3A), it possesses two technical disadvantages. First, the BS-seq fails to distinguish between m^5^C and other modified cytosine bases (e.g., hm^5^C) in mRNA (Nestor et al., 2010). Second, bisulfite treatment during sample preparation causes degradation of mRNA and thus impedes amplification of m^5^C–containing mRNA which limits the applicability of this method.

The single-molecule sequencing approach uses either of the two NGS principles. While single-molecule real-time (SMRT) technology uses nanowell (zero-mode waveguide, ZMW; Pacific BioSciences) (Vilfan et al., 2013), Nanopore sequencing (Min-ION, Oxford Nanopore Technologies) uses the change in electrical charge to detect the modified base in mRNA passing through nanopore-forming proteins (Liu et al., 2019). One of the advantages of single-molecule sequencing approaches is very long (>10,000 nt) read length, but the accuracy of the sequence is compromised. Such technologies are useful for analyzing modified bases in mRNA, particularly in a context-specific manner, as these methods allow direct sequencing of mRNA without converting it into DNA (such conversion causes the loss of modified base). However, these technologies require extensive optimization for their use in the detection of modified bases in RNA. The first experimentation on using reverse transcriptase (instead of a DNA polymerase) as the enzyme in ZMW of SMRT for direct sequencing of modified bases in mRNA was carried out by Vilfan et al. (2013). The entry of a modified base (m^6^A), present in the template mRNA, into the nanowell (ZMW) causes increased ‘inter-pulse duration’ (IPD) compared to its unmodified counterpart (adenosine). The potential of SMRT sequencing to detect m^6^A was demonstrated by Vilfan et al. (2013). Similarly, Nanopore sequencing has successfully been used to detect m^6^A in native RNA (Liu et al., 2019). Further optimization of the single-molecule sequencing technologies will revolutionize epitranscriptomic research on modified bases in mRNA.

Recently, Fang et al. (2020) reported a new method *C*RISPR *i*ntegrated *g*RNA *a*nd *r*eporter sequencing (CIGAR -seq) by combining pooled CRISPR screen and the reporters associated with RNA modification. Using the CIGAR-seq method, they could discover NSUN6 as a novel m^5^C methyltransferase in mRNA. Subsequently, they could demonstrate that this method can be successfully used to identify the regulators of other mRNA modifications such as m^1^A.

### Antibody-Based Methods for Detection of Modified Bases

RNA base modification, particularly m^6^A, is a widespread epitranscriptomic change that influences nearly every aspect of mRNA biology. Our understanding of the RNA base modification has been facilitated by the recent developments in the use of an antibody to immunoprecipitate RNAs containing modified base, and the high-throughput sequencing technologies. Methyl-RNA-immunoprecipitation-sequencing (MeRIP-seq) (Meyer et al., 2012) and m^6^A-seq (Dominissini et al., 2012) use immunoprecipitation with the help of modification (m^6^A or m^5^C)−specific antibody followed by sequencing (Figure 3B) (Yang Y. et al., 2019). Similarly, hMeRIP-seq relies on the anti-hm^5^C antibody to detect hm^5^C in Drosophila mRNA (Delatte et al., 2016). acRIP-Seq uses an ac^4^C-specific antibody to identify 4,000 ac^4^C in the human transcriptome (Arango et al., 2018). However, these antibody-based detection methods cannot detect hm^5^C and ac^4^C at single-base resolution, but the success in the single-base resolution of m^6^A by sequencing might help to optimize the method (Yuan et al., 2019). This enables studying the dynamics of epitranscriptome, a post-transcriptional regulatory mechanism for gene expression. The modification-specific antibody is used for enrichment/collection of the sample with the fragments containing the modified base. To detect m^5^C in mRNA, bisulfite-based technique cannot be used with much success. Hence m^5^C RNA immunoprecipitation (m^5^C-RIP) was used (Figure 3C) by Edelheit et al. (2013). In this method, an anti-m^5^C antibody is used to immunoprecipitate and enrich the modified base containing mRNA fragments, followed by library preparation and sequencing. Methylation individual-nucleotide-resolution crosslinking and immunoprecipitation’ (miCLIP) was used (Figure 3D) to identify m^5^C in RNA (Hussain et al., 2013; Khoddami and Cairns, 2013). This approach exploits the enzymatic activity of m^5^C methyltransferase containing a cysteine-to-alanine mutation (C271A) in NSUN2 which inhibits release of the enzyme from protein–RNA complex. This results in a covalent bond between the enzyme and its RNA targets. Antibody specific to the RNA-bound protein is used to immunoprecipitate the fragments containing the modified base, followed by library preparation and sequencing. The immunoprecipitation allows detection of methylated bases/RNAs in low-abundance without the need of deep sequencing. Subsequently, miCLIP was used to map m^6^A at single-base resolution (Linder et al., 2015).

### Antibody-Free Sequencing Methods

Many of the RNA base modification detection methods rely on the use of antibodies for immunoprecipitation. However, an antibody may fail to distinguish between two different modified forms of a nucleobase, such as m^6^A and m^6^Am. Moreover, the methods are dependent on the specificity of the antibody, which emphasizes the desire for the antibody-free method to draw transcriptome-wide atlas of the modified base. Hong et al. (2018) developed an antibody-independent method to detect m^6^A at single-nucleotide resolution via 4SedTTP incorporation and FTO demethylation. Since the 4SedTTP stably base pair with A but cause truncation on m^6^A-T pairing during reverse transcription. The RT stop signals of RNA with/without FTO treatment is then compared to determine the exact sites of m^6^A. Recently, endoribonuclease-based RNA digestion with m^6^A-sensitive RNase (*Maz*F to cleave RNA at unmethylated ACA motifs) followed by sequencing (MAZTER-seq) (Garcia-Campos et al., 2019), and m^6^A-sensitive RNA-endoribonuclease-facilitated sequencing (m^6^A-REF-seq) (Zhang Z. et al., 2019) methods were used as antibody-independent methods. Another antibody-free m^6^A sequencing (deamination adjacent to RNA modification targets, DART-seq) method was devised, using APOBEC1-YTH (cytidine deaminase fused with m^6^A-binding YTH domain) protein which deaminates C to U at the site adjacent to m^6^A. This helps to identify m^6^A sites in mRNA (Meyer, 2019). Moreover, two chemical labeling methods *viz*. m^6^A-label-seq (Shu et al., 2020), and m^6^A-SEAL (Wang et al., 2020) have also been developed. Wang et al. (2020) combined dithiothreitol (DTT)-mediated thiol-addition reaction [that converts the unstable hm^6^A to stable *N*^6^-dithiolsitolmethyladenosine (dm^6^A)] with FTO-mediated enzymatic oxidation of m^6^A to hm^6^A to develop FTO-assisted m^6^A selective chemical labeling (m^6^A-SEAL) method for detection of m^6^A in mRNA. In a transcriptome-wide m^6^A-SEAL-seq analysis, they could identify 8,605 m^6^A in human embryonic kidney and 12,297 m^6^A in rice leaf. Currently, most of the epitranscriptomic studies employ a detection method with NGS technology for context-specific mapping of the modified base at single-base resolution.

## Challenges in the Detection of Modified Bases

A major challenge in detection of the modified mRNA base has been the relatively low count of the modified base within the vast mRNA repertoire Another challenge is the precise quantification and mapping of modified RNA residues at a single-nucleotide level. Additional challenge stems from substantial background signals often present in the maps prepared. The inability of the technique to discriminate between misincorporation/RT-stop due to the modified base and background-pause/misincorporation either due to RNA structure, RT-error, or technical errors of sequencing platform (Schwartz and Motorin, 2017). Since the same antibody can recognize both m^6^A (in RNA) and 6-mA (in DNA), the contaminating DNA must be removed to get the real level of the modified base (Liang et al., 2020). Besides, there are many other limitations including intrinsic bias on secondary structures. For example, the m^6^A specific-antibody fails to distinguish between m^6^A and m^6^Am (Schwartz et al., 2013; Linder et al., 2015). Although CMC-based Ψ sequencing has been successful in identifying Ψ at the single-base resolution, it has been associated with the problem of RNA degradation because of the alkaline treatment step (see Zhao et al., 2020). Moreover, current sequencing technologies have not been able to detect hm^5^C and m^1^A, particularly at the single-base resolution, which limits the functional characterization of these modified bases. Some of the challenges in the detection of modified RNA bases at technological, experimental, and analytical levels are described here.

Sequencing by synthesis approach has many restrictions in detecting the base modification. The specific antibody or chemical required for the detection of a modified base (indirect detection of the modified base) is known for only a limited number of modifications, which may show cross-reactivity. The antibody-based immunoprecipitation (IP) sequencing method (e.g., m^6^A-seq or MeRIP) uses a 100–200 nt mapping window which fails to precisely identify m^6^A sites (Molinie et al., 2016). The photocrosslinking-assisted m^6^A sequencing (PA-m^6^A-seq), m^6^A individual-nucleotide-resolution crosslinking and immunoprecipitation (miCLIP), and UV-CLIP techniques suffer from low crosslinking yield and use an indirect method to infer m^6^A sites. The location of m^6^A is inferred near the antibody crosslinking point (the tyrosine residue of antibody and RNA base), but the crosslinking point might be at varying distance from the m^6^A-binding sites, which creates difficulty in precise identification of the m^6^A site, particularly when m^6^A occurs in a cluster (Meyer et al., 2012; Linder et al., 2015). Even the direct detection methods like SMRT face certain challenges such as the ZMW stumbles when a stretch m^6^A gets incorporated; hence, the current throughput level is too low for transcriptome-wide analysis.

Careful selection/inclusion of input controls for base modification mapping is crucial. Mostly, well-known modified bases in rRNA/tRNA serve as intrinsic controls for evaluating the sensitivity and specificity of analytical methods. The conventional transcriptome analysis uses millions of cells from a tissue, epitranscriptome being highly dynamic, the cell-specific analysis would be necessary for the detection/quantification of a particular base modification and its functional characterization (Helm and Motorin, 2017). Furthermore, biological and/or technical replicates (at least 2–3) are very important to filter out falsely-detected sites, as well as to assess the robustness/reproducibility of the detection and quantification method.

## Future Perspectives

Recent studies have provided unprecedented mechanistic insights into RNA base modifications, and NGS-based technologies for detecting RNA base modifications are further improving the scenario. Modern chemical biology tools would be applied to expedite the epitranscriptomic studies. High-throughput technologies to simultaneously identify different modified bases in the same RNA molecule will have considerable applicability, as modified bases may have cumulative effects on regulating biological functions. Plants provide a unique system to elucidate the biological functions of modified RNA bases and their regulatory aspects through investigating epitranscriptomic alterations in higher eukaryotes, which are otherwise difficult to be elucidated using an animal system (Shen et al., 2019). Using a combination of techniques including genetic ablation and NGS-based mapping, the regulatory roles of the epitranscriptome in several developmental processes in plants have been demonstrated. Compared with the writers and erasers, readers for the modified base play a more significant role in responses to environmental stresses. This suggests that deciphering the location/context of epitranscriptomic marks is more important than merely detecting the changes (writing/erasing) in the marks for improved stress adaptation in plants. Therefore, it is important to characterize the role of reader proteins in the epitranscriptomic regulation of gene expression under environmental stresses (Hu et al., 2019). Association between epitranscriptome and stress responses in plants indicates that such epitranscriptomic marks might be utilized in the future as important epimarks for the development of stress-tolerant crop plants (Vandivier and Gregory, 2018). In the line of the success in the detection of m^6^A at single-base resolution, a similar sequencing method would be optimized for hm^5^C and m^1^A mapping. Nevertheless, for functional characterization of epitranscriptomic modifications, quantification of the absolute stoichiometry of RNA modifications is crucial.

However, several questions need to be answered before we can devise appropriate strategies to better utilize the epitranscriptomeic information. Some of these include, why only selected mRNAs get modified? Why are only certain adenosine/m^6^A or cytosine/m^5^C at selected sites gets methylated/demethylated? How does the modified base affect downstream mRNA processing? How do different readers recognize their targets? How are the writer, reader, and eraser for a nucleobase get co-ordinately regulated by the developmental/environmental signal? Even if we get answers to some of these questions, several other questions would require to be answered. For example, how do the different mRNA base modifications influence the dynamics/function of each other? Experiments designed to answer some of these questions are underway in laboratories worldwide, and we expect that the next 5 years of research in epitranscriptomics would be more exciting than the past!.

## Conclusion

During the past few years, many RNA modifications and their functional versatility could be discovered due to the advances in chemogenetic RNA labeling techniques, high-throughput NGS, and functional validation. Several other dynamic base modifications in mRNA are also being identified, which would require functional characterization for advances in epitranscriptomics. Numerous other epitranscriptomic modifications may be identified in the future which may show interaction with other modified bases in modulating metabolic pathways. The biological functions of several mRNA base modifications are still poorly understood, their detection at single-base resolution using technological advancements such as nanowell (SMRT) and nanopore (Oxford Nanopore) sequencing is very much promising. However, proper experimental design with a sufficient number of replications, and inclusion of controls would be very important to rule out false-positive results and for the highest confidence level. Moreover, identifying the enzyme(s) involved in modification of RNA base (reader), and replacing it with an unmodified base (eraser) is necessary for devising strategies to manipulate the expression of a gene. However, several fundamental questions remain to be answered, including whether modified bases are conserved among plant species. Answering these questions would substantially improve our knowledge of epitranscriptomics and its effects on plant growth, fitness, and survival under environmental stress. Such investigations, particularly comprehensive studies to demonstrate a linkage between epigenetic and epitranscriptomic regulations, would offer potential new strategies for the manipulation of crop plants with better plasticity/adaptability to the changing climatic conditions. Comprehensive studies on the correlation between epigenetic and epitranscriptomic regulation of gene expression might provide some newer aspects (Song and Yi, 2017) for the manipulation of a trait through epigenome/epitranscriptome editing to develop climate-smart crop plants for the 21st century (Kumar, 2019).

## Author Contributions

SK and TM conceived the review. SK prepared the manuscript. SK and TM revised the manuscript and approved the final draft. Both authors contributed to the article and approved the submitted version.

## Conflict of Interest

The authors declare that the research was conducted in the absence of any commercial or financial relationships that could be construed as a potential conflict of interest.
